# Genome-Wide Association Mapping of Ear Traits in Early-Maturing Maize Under Contrasting Planting Densities

**DOI:** 10.3390/ijms27188259

**Published:** 2026-09-16

**Authors:** Lijing Jiang, Shuaiheng Chen, Yuan Gao, Tieqiang Zhang, Hui Wang, Renjie Wang, Anbo Zheng, Minxu Jiang, Yi Wang, Hongwei Zhang

**Affiliations:** 1 Economic Crops Research Institute, Heilongjiang Land Reclamation Academy of Sciences, Harbin 150030, China; 2 College of Informatics, Huazhong Agricultural University, Wuhan 430070, China; 3 Department of Bioengineering, Harbin Institute of Technology, Weihai 264209, China; 4 Institute of Crop Sciences, Chinese Academy of Agricultural Sciences, Beijing 100081, China

**Keywords:** high-density planting, candidate gene, cold-adapted maize, genetic diversity, selection signature

## Abstract

Developing early-maturing maize varieties with improved tolerance to high-density planting is essential for enhancing yield in China. In this study, a unique panel of 498 cold-adapted early-maturing inbred lines were evaluated for ear length (EL) and ear diameter (ED) across two locations under contrasting planting densities in one growing season. Phenotypic analysis revealed abundant phenotypic variation in these ear traits among the tested lines, and there were significant correlations between environments. A total of 406,897 high-quality single nucleotide polymorphism markers (SNPs) were obtained from liquid chip analysis. Population structure analysis classified the early-maturing maize germplasm into 9 distinct genetic groups, with obvious genetic differentiation between groups. Linkage disequilibrium (LD) analysis demonstrated rapid LD decay and high genetic variation in the population. A genome-wide association study (GWAS) identified 380 quantitative trait loci (QTLs) for both ED and EL under two planting densities. In total, 138 and 89 annotated genes were identified within QTLs detected under normal and high-density planting conditions, respectively. Among them, *Zm00001eb288330* (a TPX2 family protein) and *Zm00001eb366770* (an OVATE family transcription factor), which were located close to the top SNPs for ED and EL under high planting density, were considered positional candidate genes based on their annotations. Furthermore, by integrating candidate gene analysis with selection signatures, we found that candidate genes detected under high-density planting conditions show signatures of ongoing selection during maize breeding. These findings identify candidate genes for future functional validation and breeding-oriented follow-up studies.

## 1. Introduction

Maize (*Zea mays* L.) serves as a pivotal source of food, feed, and biofuel. In addition to temperate and tropical regions, maize can be cultivated in cold-temperate zones, where the active accumulated temperature can be as low as 1900 °C [1]. The development of cold-adapted maize varieties capable of achieving economic yield within a shorter growing season is very important for both farmers and society in cold-temperate zones. A key modern breeding strategy to maximize yield potential is the adoption of high-density planting, which increases the number of plants per unit area [2]. However, under dense planting conditions, plants experience intensified competition for light, water, and nutrients, leading to increased barrenness, stalk lodging, and the deterioration of ear traits that directly determine yield.

In maize, the ear is a critical yield-determining organ. Ear traits are complex quantitative characteristics controlled by several interconnected gene regulatory networks. The most extensively characterized is the *WUSCHEL* (*WUS*)-*CLAVATA* (*CLV*) signaling feedback loop [3], which maintains the stem cell population in the inflorescence meristem. *ZmWUS1* and *ZmWUS2* promote meristem activity, while their expression is negatively regulated by the secreted peptides ZmCLE7 and ZmFCP1, perceived by receptors including TD1, FEA2, and FEA3. Disruption of this loop leads to fascinated and enlarged ears [4,5,6]. Another key module involves the bZIP transcription factor FEA4 and the glutaredoxin MSCA1; FEA4 modulates meristem determinacy and ear size, and both FEA4 and MSCA1 interact with ZmWUS proteins [7,8]. The development of spikelet pair meristems (SPMs) relies on the SBP-box transcription factors tsh4, ub2, and ub3, with ub3 being quantitatively regulated by the distant enhancer *KRN4*, a major QTL for kernel row number [9,10,11]. Collectively, these pathways provide a genetic framework for understanding ear trait variation under high-density planting conditions.

High-density planting becomes a main strategy to increase maize grain yield. Research on the genetic regulation of high-density planting has primarily focused on plant architecture. Extensive genetic studies have uncovered key regulators for architectural traits. For instance, plant height is mainly governed by phytohormone pathway genes [12], while leaf angle is shaped by genes controlling ligule–auricle morphogenesis, hormone signaling, midrib mechanical tissue development, and gravity responsiveness [13]. Improvement in these architectural traits enhances lodging resistance, canopy light capture and ventilation, and photosynthetic efficiency, and optimizes assimilate partitioning between source and sink organs, thereby influencing ear traits and boosting population-level productivity [2]. However, genetic mapping of ear traits under high-density planting has not yet been reported.

Ear development is highly sensitive to environmental factors, including cold stress and high-density planting stress [14,15]. Under the combined pressure of cold and high-density planting, dissecting the genetic basis of ear traits is critically important. Although genome-wide association studies (GWASs) have successfully elucidated the genetic architecture of numerous agronomic traits in temperate maize, their application to cold-adapted maize germplasm under high-density stress remains limited. Populations composed of cold-tolerant inbred lines likely harbor novel allelic variations and adaptive genes selected for low-temperature tolerance [16], which may interact with pathways related to high-density planting in manners not detected in conventional germplasm.

This study aims to bridge this knowledge gap by performing a comprehensive GWAS on major ear traits in a special panel of cold-adapted maize inbred lines under high-density planting conditions to identify condition-specific associations. Utilizing high-density SNP markers, we seek to identify significant marker–trait associations and identify genomic regions associated with the phenotypic variation of these traits. Beyond mere locus identification, a primary objective is to conduct in-depth candidate gene mining and investigate whether they are selected during maize breeding. The findings from this research are expected to provide novel insights into the genetic mechanisms controlling ear development under the combined constraints of cold and high-density planting.

## 2. Results

### 2.1. Phenotypic Data Analysis

**Figure 1:**
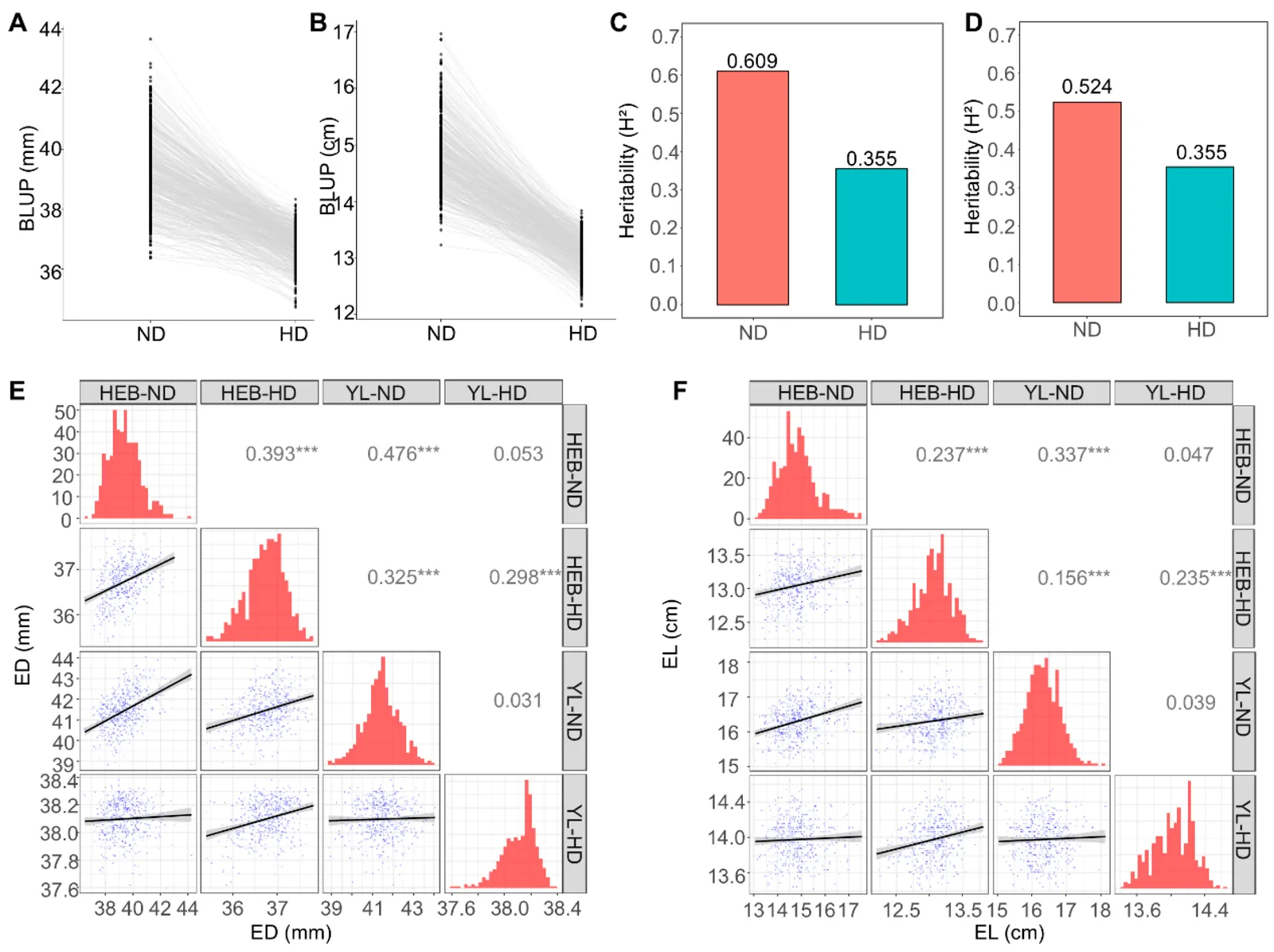
Phenotypic data analysis of ED and EL under two planting densities. Note: (**A**) ED and (**B**) EL showed a phenotypic relationship between ND and HD planting, where ND and HD indicate normal and high densities, respectively. (**C**,**D**) are the broad-sense heritability for ED and EL, respectively. In (**E**,**F**), the diagonal displays the phenotypic distribution of each trait. Above the diagonal, we present the correlation coefficients, with *** denoting *p* < 0.001 and the absence of asterisks indicating non-significant correlations. Below the diagonal, we show scatter plots with fitted regression lines for the corresponding trait pairs. ED, ear diameter; EL, ear length; ND, normal density; HD, high density; HEB, Harbin site; YL, Yilin site.

Under normal-density planting, ED ranged from 37.4 mm to 44.7 mm, and EL ranged from 13.8 cm to 18.0 cm. Under high-density planting, ED ranged from 35.4 mm to 39.0 mm, whereas EL ranged from 12.6 cm to 14.3 cm (Figure 1A,B; Table S1). Notably, both ED and EL exhibited wider phenotypic variation under normal planting compared with high-density planting, indicating that high-density conditions constrained the range of phenotypic variability (Figure 1A; Table S2). Consistent with previous studies reporting that stress reduces phenotypic stability [17], the heritability of both traits under normal planting was higher than that under high-density planting (Figure 1C,D). The reliability of the phenotypic dataset was further validated by environmental correlation analyses. For ED, the correlation coefficients between the two locations were 0.476 (normal density) and 0.298 (high density) (Figure 1E); for EL, they were 0.337 (normal density) and 0.235 (high density) (Figure 1F). These moderate correlations indicate that ear traits are influenced by location and possibly by genotype-by-location interaction, particularly under high-density stress.

### 2.2. Genotypic Data Analysis

**Figure 2:**
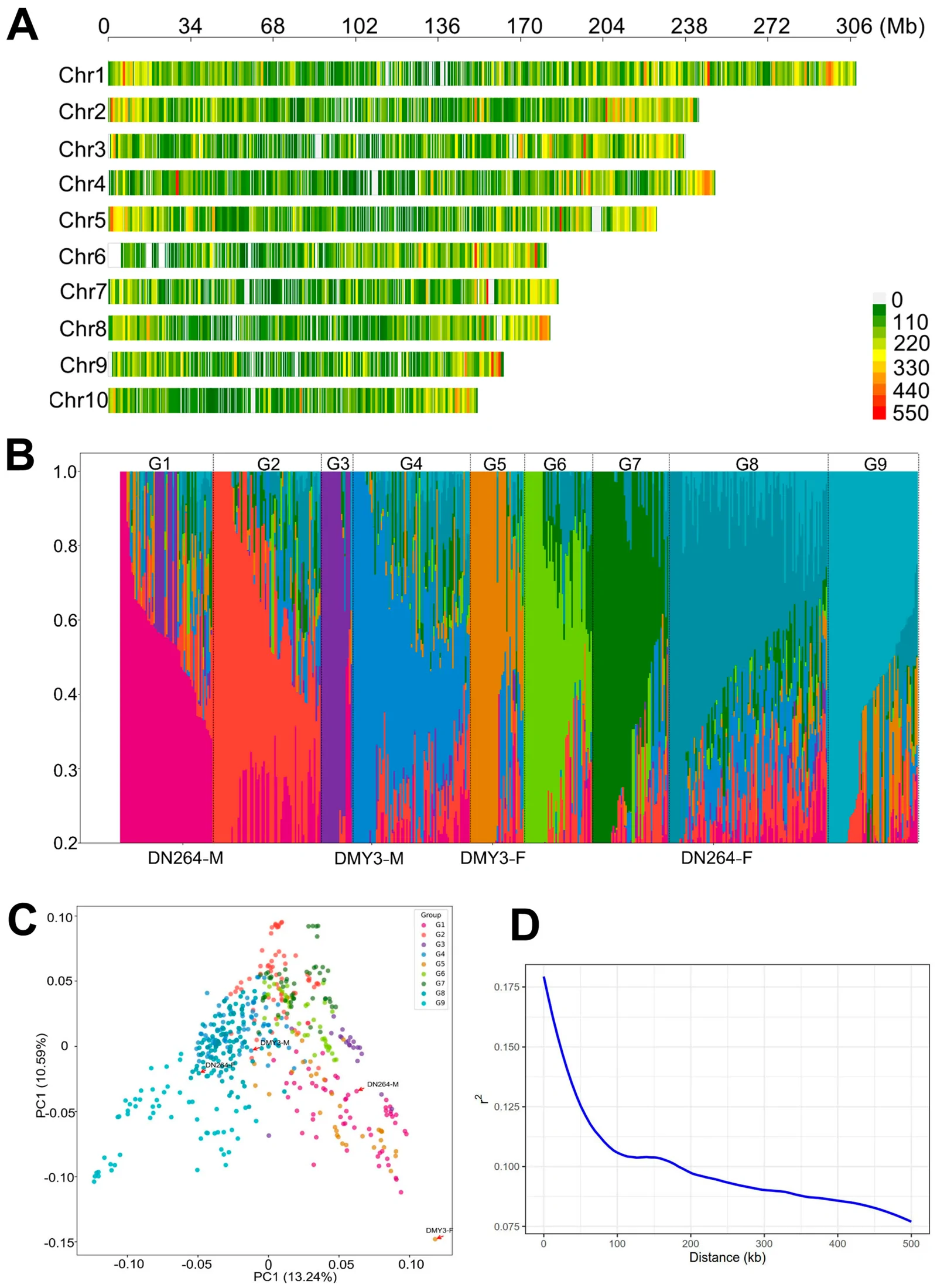
Genotypic data analysis. Note: (**A**) Distribution of SNPs across the ten chromsomes; (**B**) Classification of the population into nine groups; (**C**) PC analysis of the population; (**D**) LD analysis of the population. DN264 (dongnong264) and DMY3 (demeiya3) are popular early-maturing varieties in cold-temperate zones of Northern China. M and F indicate male and female, respectively.

A total of 406,897 SNPs were detected and were distributed evenly across the chromosomes (Figure 2A). Population structure analysis divided these lines into nine groups (Figure 2B). DN264 and DMY3 are very popular in early-maturing varieties in cold-temperate zones of Northern China. The male and female parents of DN264 and DMY3 are in different groups. By performing a PC (principal component) analysis, we found that PC1 and PC2 can explain 13.24% and 10.59% of the variance, and the nine groups distribute in a cluster in the PC plot (Figure 2C). The LD analysis showed that the LD decay reached a steady level at 100 kb (Figure 2D). Genotypic data revealed that the population showed great genotypic diversity and rapid LD decay.

### 2.3. GWAS of ED and EL Under Two Planting Densities

**Figure 3:**
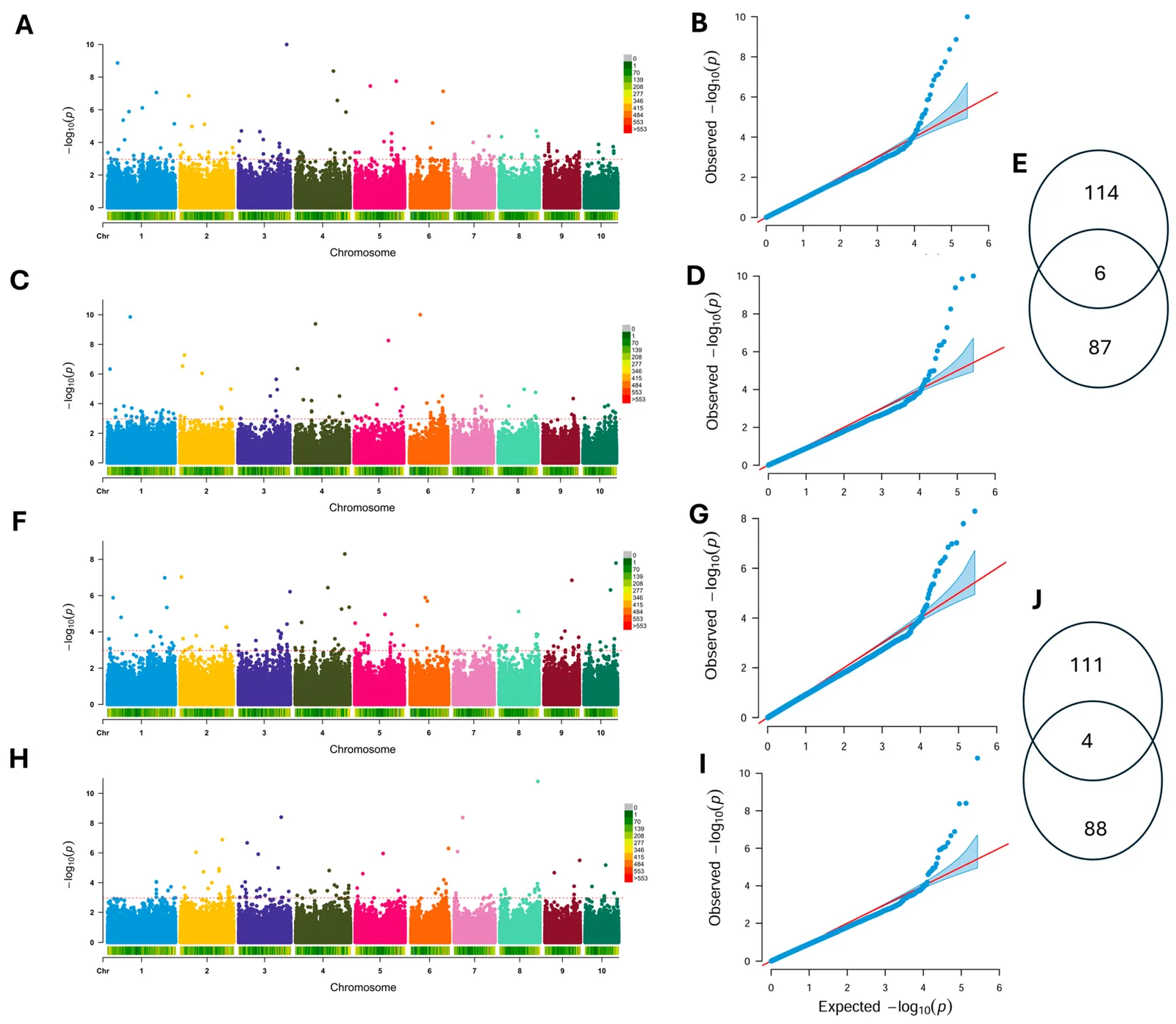
GWAS of ED and EL under both planting densities. Note: (**A**,**B**) GWAS for ED under normal planting density. (**C**,**D**) GWAS for ED under high planting density. (**E**) Common QTLs detected for ED under both planting densities. (**F**,**G**) GWAS for EL under normal planting density. (**H**,**I**) GWAS for EL under high planting density. (**J**) Common QTLs detected for EL under both planting densities.

A GWAS was performed for ED under two planting densities (Figure 3A–D), and the QQ plot indicates that false positives were properly controlled despite cryptic genetic relatedness (Figure 3B,D). The GWAS identified 166 significant SNPs under normal planting density, which were clustered into 114 QTLs, with 61 unique genes annotated within 83 QTL regions (Figure 3A; Table S3). Under high-density planting, the GWAS detected 131 significant SNPs corresponding to 87 QTLs, and 45 unique genes were found in 66 QTL regions (Table S4). In total, 6 consensus QTLs for ED were detected under both normal and high-density conditions (Figure 3E; Table S5). Among these, one positional candidate gene was identified for a common QTL on chromosome 10, *Zm00001eb071940*, which was annotated as a P-loop containing nucleoside triphosphate hydrolase superfamily protein.

A GWAS was also performed for EL under both planting densities (Figure 3F–I), and the GWAS identified 150 significant SNPs under normal planting density, which were clustered into 111 QTLs (Figure 3F,J), with 67 unique genes annotated within 91 QTLs regions (Table S6). Under high-density planting, 131 significant SNPs corresponding to 88 QTLs were detected, and 44 unique genes were associated with 72 of these SNPs (Figure 3J; Table S7). A total of four consensus QTLs for EL were identified across both densities (Table S8), one of which harbored the positional candidate gene *Zm00001eb403370*, encoding a plant-specific secretory peptide.

### 2.4. Candidate Gene Association Mapping and Single-Locus Genotype Analysis

**Figure 4:**
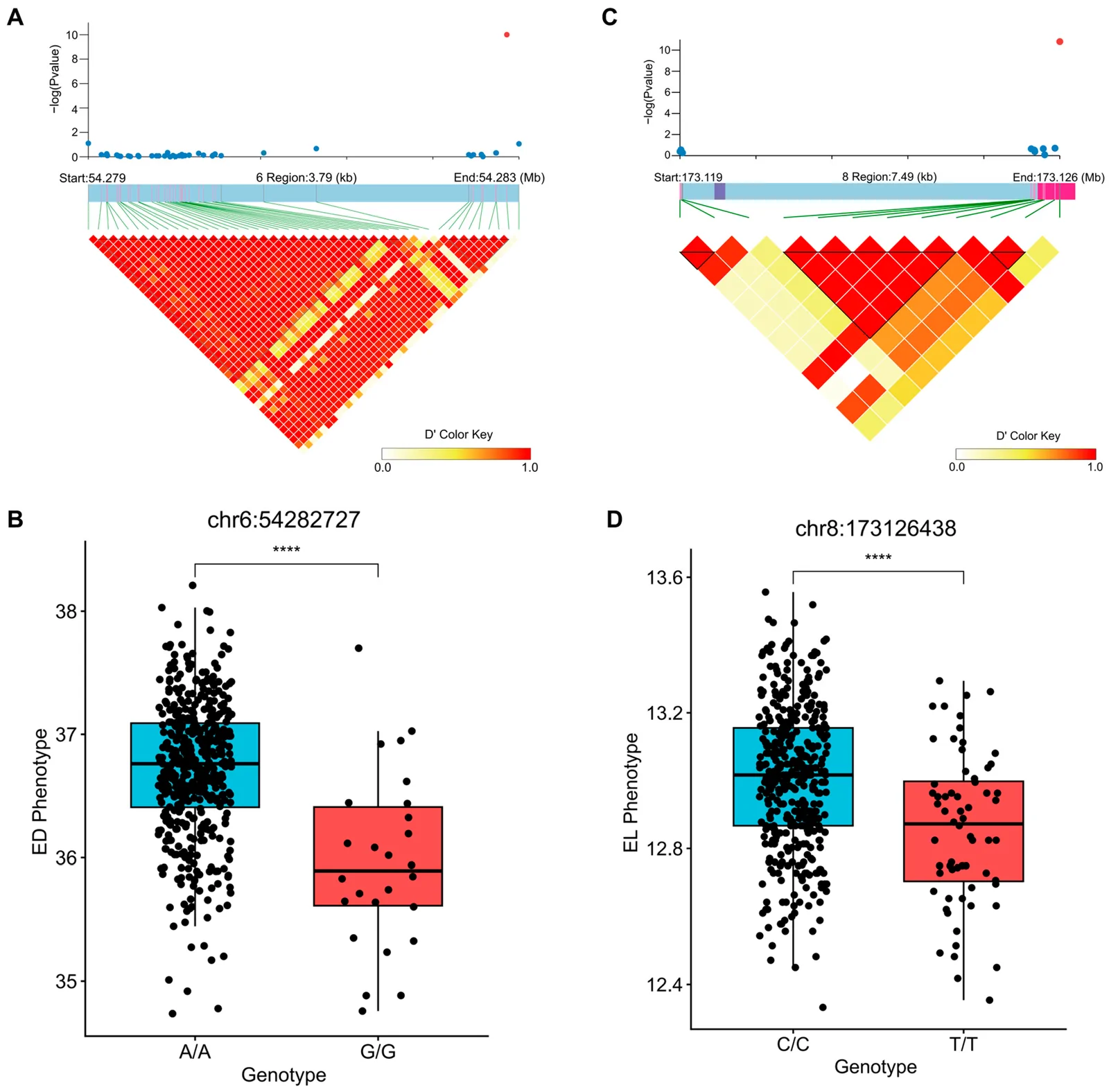
Two prioritized loci were supported by local association patterns and showed clear genotype-dependent differences in ED and EL under high-density planting. (**A**) Regional plots around the lead SNPs on Chr.6 are associated with ED under high-density planting. Using the genome-wide LD-decay estimate, *Zm00001eb288330* was retained as a positional candidate. (**B**) Single-locus genotype comparisons were performed using the lead SNPs on chr.6. (**C**) Regional plots around the lead SNPs on chr. 8 are associated with EL under high-density planting. Using the genome-wide LD-decay estimate, *Zm00001eb366770* was retained as a positional candidate. (**D**) Single-locus genotype comparisons were performed using the lead SNPs on chr.8. **** denotes significant pairwise differences at the *p* < 0.0001 level.

Regional association analysis narrowed the candidate intervals around two top SNPs for ED and EL under high-density planting, respectively (Figure 4; Tables S4 and S7). Using an LD-supported window on either side of each lead SNP, we found one positional candidate gene for ED and one for EL in the Zm-B73-REFERENCE-NAM-5.0 assembly, respectively: *Zm00001eb288330* and *Zm00001eb366770*. According to the B73 Zm00001eb.1 annotation, *Zm00001eb288330* encodes a TPX2 (targeting protein for Xklp2 2) family protein, and these family members were associated with responses to multiple abiotic stresses [18]. *Zm00001eb366770* encodes an OVATE-transcription factor family protein (OFP), and this family is essential in regulating response to abiotic stresses in plants [19,20]. These annotations indicate that these genes may be related to resistance to high-density planting, an important abiotic stress. Based on the genotypes of the lead SNPs at these two loci (Tables S4 and S7), single-locus genotype comparisons showed significant differences in ED and EL between genotype classes at the two prioritized SNPs (Figure 4B,D). These results highlight two positional candidate genes and linked variants for maize high-density breeding.

### 2.5. Temporal Dynamics of Genetic Diversity of Candidate Genes Detected Under High-Density Planting Conditions

**Figure 5:**
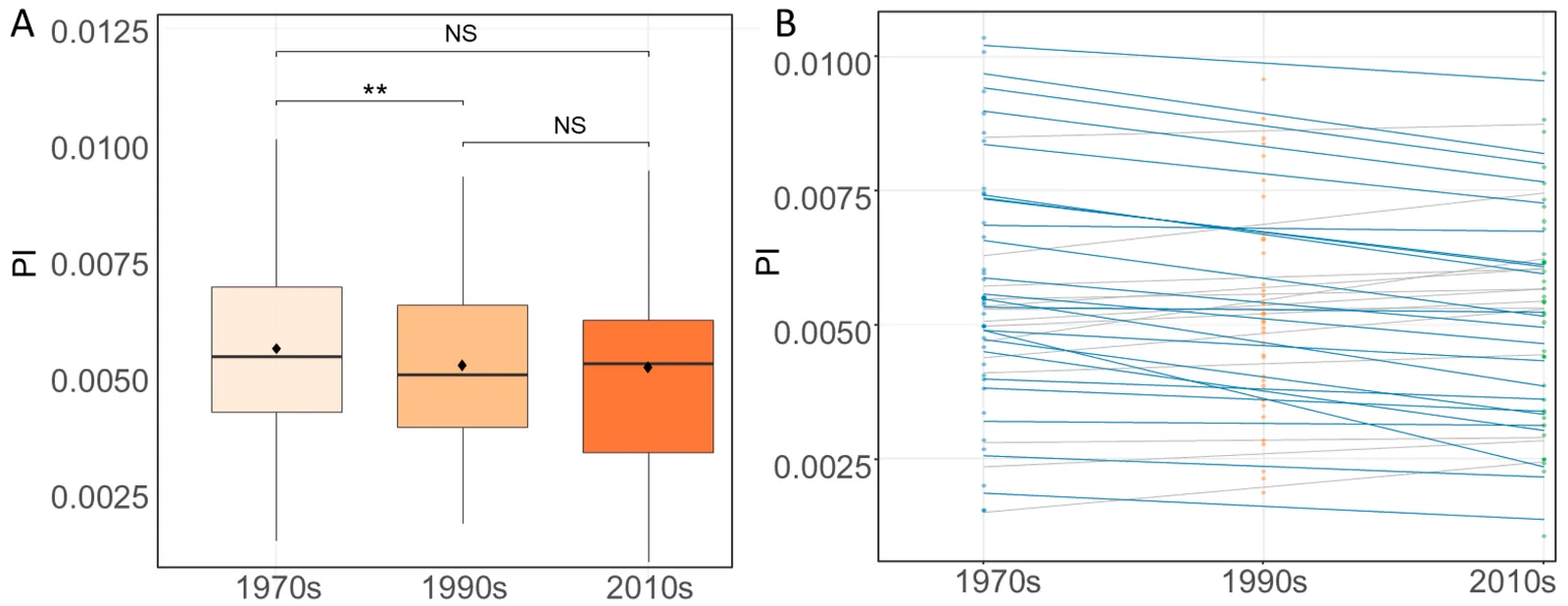
Temporal dynamics of genetic diversity of candidate genes detected under high-density planting conditions. (**A**) The Pi values of 89 candidate genes in different breeding eras. “NS” and “**” denotes “no significant difference” and “significant pairwise differences at the *p* < 0.01 level”, respectively. (**B**) Linear regression analysis of Pi values of candidate genes.

To dissect the selection dynamics of candidate genes for these candidate genes during the genetic improvement of maize in China, we conducted a systematic analysis of temporal trends in the genetic diversity of these genes using breeding materials, which represents different breeding eras in China. The diversity of the 89 candidate genes underlying QTLs detected for both ED and EL under high planting density showed a significant reduction with the progression of the breeding process (Figure 5A). Linear regression analysis revealed that the Pi values of more than 65% of the candidate genes showed a continuous downward trend with the progression of breeding eras (from the 1970s to the 2010s) (Figure 5B), indicating that most candidate genes showed patterns suggestive of ongoing selection during long-term genetic improvement.

## 3. Discussion

Compared with temperate maize, cold-temperate maize has formed a unique genetic background during long-term adaptation. It not only carries a large number of cold-resistant and early-maturing alleles but may also differ from temperate materials in the genetic mechanisms underlying traits detected under high-density planting [21]. This study focuses on a unique panel of cold-adapted, early-maturing inbred lines from northern China, a germplasm pool that has been largely uncharacterized in prior studies. Due to the short growing season typical of cold-temperate regions and the limited availability of uniform field sites for such a large panel, our study was conducted at two representative locations with two replications. Although fewer than three environments, the combination of two locations × two planting densities effectively provided four distinct environmental conditions, strengthening the assessment of genotype-by-environment interaction. Significant phenotypic correlations between locations (Figure 1) and the identification of six consensus QTLs stable across both densities support the reliability of the detected loci. Additional environments would improve power and plan to validate the key loci in future multi-environment trials.

By dissecting the genetic basis of ear traits in early-maturing maize in cold-temperate zones of Northern China under contrasting planting densities, this study dissected the genetic basis of maize ear traits with an emphasis on high-density stress, filling the gap in genetic research on traits under high-density planting in this ecological region. The short growing seasons in Northern China indicate that maize breeding must balance early maturation and high yield, and high-density planting is an important approach to achieving high yield. This panel has accumulated abundant genetic variation adapted to high-density planting through decades of natural selection and artificial domestication, which directly addresses breeding needs for high-density planting in cold regions. These QTLs are candidates for further validation in independent populations before any breeding application can be considered.

Focusing on ear traits under high-density planting is a key aspect of this study. Yield losses caused by high-density planting mainly result from the deterioration of ear traits, such as a shortened ear length, a reduced ear diameter, and an increased barren tip length [22]. Most previous genetic studies on high-density planting have focused on lodging-related traits, such as plant height and stem diameter [23,24], while research on ear traits that directly determine yield under high density is relatively insufficient. This study analyzed the phenotypic variation and genetic structure of ear length and ear diameter under two planting densities and found that the heritability of ear traits under high-density conditions was significantly reduced, indicating that they are more affected by the environment. By adopting multi-environment trials and the FarmCPU multi-locus association analysis model, we identified 6 cross-density stable QTLs for ear diameter and 4 for ear length and discovered a batch of QTLs that are specifically detected under high density. The identification of these genomic loci detected under high-density planting conditions provides key targets for breeding maize varieties that can maintain excellent ear traits under dense planting conditions.

We prioritized two positional candidate genes, *Zm00001eb288330*, encoding a TPX2 family protein, and *Zm00001eb366770*, encoding an OVATE family transcription factor (OFP), that were significantly associated with ED and EL under high-density planting conditions (Figure 4). High-density planting imposes a complex array of interconnected abiotic stresses on maize, including altered light quality (a low red-to-far-red ratio), limited light penetration, intensified root competition for water and nutrients, and mechanical impedance. These factors collectively reduce phenotypic stability and heritability, as observed in our study (Figure 1). The identification of TPX2 and OFP family members as candidate determinants offers an entry point to dissect the molecular mechanisms by which plants coordinate development and stress adaptation under agronomically relevant adversity. TPX2 family members associate with cortical microtubules and are profoundly influenced by drought, salinity, and extreme temperatures [2]. In the context of high-density planting, the TPX2 family protein may preserve the integrity of the microtubule network in cob and kernel tissues. OFPs have subsequently emerged as versatile modulators of organ shape, secondary cell-wall deposition, and hormone signaling [16]. Functional studies across diverse species have established that OFPs are pivotal in regulating responses to drought, salinity, and osmotic stress [19,20,25,26] and may act as a negative regulator of shade-induced growth, likely by repressing hormone-responsive cell elongation genes. However, transgenic or gene-editing experiments are required to confirm their roles in high-density planting.

This study integrates candidate gene mining with selection signature analysis, revealing that loci detected under high-density planting have been subjected to directional selection during maize improvement. Since the 1970s, the increase in global maize yield has mainly benefited from the increase in planting density [27], and the genetic improvement in density tolerance is a core objective for achieving high yield. In this study, we found that the genetic diversity of more than 65% of the candidate genes detected under high-density planting conditions showed a continuous downward trend. This result indicates that during the past half-century of maize breeding in China, breeders have been conducting directional selection on genes associated with high-density planting, leading to a continuous enrichment in their favorable alleles in modern varieties and a gradual reduction in genetic diversity. This finding verifies that high-density planting is a core target trait of maize breeding.

## 4. Materials and Methods

### 4.1. Phenotypic Evaluation and Analysis

A collection of 498 inbred lines (population 1) from cold-temperate zones in Northern China were used in this study (Table S1). We planted these inbred lines in two locations, Harbin (45°45′ N, 126°37′ E) and Yilan (46°19′ N, 129°34′ E), both in Heilongjiang Province, in the summer of 2024. Field experiments were conducted in the summer of 2024 at two locations in Heilongjiang Province, China: Harbin (45°45′ N, 126°37′ E) and Yilan (46°19′ N, 129°34′ E). Sowing was performed on May 10th and 12th of 2024, respectively, in Harbin and Yilan, and harvesting took place in late September 2024, when the plants reached physiological maturity. At each location, the inbred lines were planted in single-row plots, with a plot length of 3 m. A randomized complete block design with two replications was employed across all environments. Two planting densities were implemented: a normal density of 75,047 plants ha^−1^ (corresponding to a row spacing of 65 cm and a plant spacing of 20.5 cm) and a high density of 133,779 plants ha^−1^ (row spacing of 65 cm and plant spacing of 11.5 cm). Fertilizers were applied as follows: a compound fertilizer (N-P_2_O_5_-K_2_O; 15-15-15) at 750 kg ha^−1^ was applied as a basal fertilizer before sowing, and urea (46% N) at 225 kg ha^−1^ was applied at the V6 stage. At maturity, the ears from the middle five plants within each plot were harvested. Subsequently, ear length (EL) and ear diameter (ED) were measured and recorded for subsequent analysis.

For each planting density separately (normal and high density), the following mixed linear model was fitted to the phenotypic data from both locations:

$$y_{ijk}=\mu +L_{i}+R_{j\left(i\right)}+G_{k}+{\left(G\times L\right)}_{ik}+\varepsilon_{ijk}$$

where $y_{ijk}$ is the observed phenotype for genotype k in replication j within location i; μ is the overall mean; $L_{i}$ is the fixed effect of location i; $R_{j\left(i\right)}$ is the fixed effect of replication j nested within location i; $G_{k}$ is the random effect of genotype k, assumed as $G_{k}∼N\left(0,\sigma_{G}^{2}\right)$; ${\left(G\times L\right)}_{ik}$ is the random genotype-by-location interaction, assumed as $N\left(0,\sigma_{GL}^{2}\right)$; and $\varepsilon_{ijk}$ is the residual error, assumed as $N\left(0,\sigma_{\varepsilon }^{2}\right)$.

The BLUP for each genotype was then obtained from the fitted model. These BLUPs were used as the phenotype for the GWAS of each trait–density combination. The analysis was performed in R using the lme4 package.

The broad-sense heritability was estimated with the following equation:

$$H^{2}=\frac{\sigma_{g}^{2}}{\sigma_{g}^{2}+\frac{\sigma_{gl}^{2}}{e}+\frac{\sigma_{\varepsilon }^{2}}{er}}$$

where $\sigma_{g}^{2}$ is the genetic variance, $\sigma_{gl}^{2}$ is the variance of genotype by location interaction, $\sigma_{\varepsilon }^{2}$ is the error variance, and *e* and *r* are the number of environments and replicates, respectively. This model was fitted using the AOV function in the software IciMapping version 4.1 [28]. Correlation analysis was performed using version R 4.5.0 of the R software.

### 4.2. Genotypic Analysis

Leaf tissues were sampled from all the inbred lines, and DNA were extracted using the CTAB method. The DNA samples were sent to Beijing Baiyu Biotechnology Co., Ltd., (Beijing, China) for genotypic characterization, using a previously reported maize chip [29]. Rigorous quality control was performed on the original genotype data. SNPs with a call rate below 99%, loci with more than 50% missing data, and SNPs with a minor allele frequency (MAF) below 0.05 were removed. The missing genotypes were imputed using the codeGen function of the R package “synbreed”, the method “beagle” was chosen, and the other settings were default [30]. A total of 406,897 high-quality SNPs were obtained. For the main GWAS, we used a VCF file containing 500 inbred lines with 406,897 SNPs. The same genotypic data were used only for the PCA, population structure, and phylogenetic analyses. No samples were removed from the 500-line GWAS panel after quality control.

### 4.3. GWAS and Candidate Gene Identification

The GWAS analysis was performed using the rMVP package in the R language [31]. We adopted the FarmCPU (Fixed and random model Circulating Probability Unification) model, a multi-locus model that effectively controls population structure [32]. During model operation, the maximum number of cycles (maxLoop) was set to 10 to ensure convergence. The genomic inflation factors (λ) for ED under normal and high-density planting conditions were 0.8996 and 0.8414, respectively, whereas those for EL under normal and high-density planting conditions were 0.8942 and 0.8946, respectively. All λ_GC values are less than 1, indicating no obvious statistical inflation or serious false positives in the GWAS results, but the overall results are somewhat conservative. This may be related to the control of population structure and kinship by the FarmCPU model.

After quality control, a total of 278,408 effective SNPs were used for association analysis. The significance cutoff for candidate locus selection was calculated as 300/278,408 ≈ 1.08 × 10^−3^. This threshold was intended as a candidate-locus screening threshold rather than a strict genome-wide threshold, balancing sensitivity and false-positive control while allowing for approximately 300 expected false positives under the null hypothesis.

Based on the GFF3 annotation file of the maize reference genome Zm-B73-REFERENCE-NAM-5.0, gene mapping was performed for the significantly associated SNP loci. According to the genome-wide LD analysis, a genomic window of ±100 kb was constructed around each significant SNP. Gene intervals were then extracted from the GFF3 file, and BEDTools was used to identify overlaps between the SNP-extended intervals and annotated genes [33].

### 4.4. Candidate-Gene Association Analysis and Single-Locus Genotype Analysis

Candidate genes were selected from the annotated genes overlapping the lead SNP or located within LD-supporting regions of the lead SNP. On the basis of this criterion, *Zm00001eb288330* on chr. 6 was retained as the candidate gene for ED under high planting density, because this gene was close to the top SNPs that had the largest −log(P) value. Meanwhile, Zm00001eb366770 on chr. 8 was retained as the candidate gene for EL under high planting density. Single-locus genotype comparisons were then performed to evaluate differences in ED and EL under high-density planting among genotype classes at the prioritized SNPs. Pairwise comparisons among genotype classes were conducted with the Wilcoxon rank-sum test.

### 4.5. Nucleotide Diversity Trends of Candidate Genes in Different Eras

Population 2, which was used for studying germplasm evolution patterns, consisted of 189 maize breeding materials from China. Based on pedigree origin and release-decade information, these lines were divided into three era groups for dynamic analysis: the “1970s and earlier” group (1970s; 44 lines), the “1980s–1990s” group (1990s; 99 lines), and the “2000s–2010s” group (2010s; 46 lines) [34]. Using the genotypic data of population 2, the Pi values of 89 genes in QTL regions detected under high-density planting conditions were calculated. For each candidate gene, Pi was calculated using the formula π = Σ(2 × p_i × q_i)/L, where p_i and q_i are the allele frequencies of the two alleles at the i-th segregating site, and L is the total length of the analyzed region. The computation was performed using VCFtools (vcftools_0.1.12) based on the genotypic data. Taking the breeding era as the independent variable and the Pi values of each era group as the dependent variable, a linear regression analysis was performed for each gene to obtain the regression coefficient.

## 5. Conclusions

A GWAS was performed for EL and ED using 498 early-maturing maize inbred lines from Northern China under normal and high planting densities. Phenotypic analysis revealed that high-density stress significantly narrowed the phenotypic variation range of ear traits and reduced heritability. Population structure analysis divided the tested materials into nine genetic groups, with rapid LD decay, making the population suitable for high-resolution association mapping. A total of 380 QTLs were detected, with 89 candidate genes being identified for QTLs detected under high-density planting. Over 65% of the candidate genes exhibited a continuous decreasing trend in nucleotide diversity during long-term breeding. This study provides important genetic resources and theoretical foundations for high-density breeding in early-maturing areas.

## Data Availability

The data are available in the Supplementary Tables.

## Supplementary Materials

The following supporting information can be downloaded at: https://www.mdpi.com/article/10.3390/ijms27188259/s1.
